# Roles of Organohalide-Respiring *Dehalococcoidia* in Carbon Cycling

**DOI:** 10.1128/mSystems.00757-19

**Published:** 2020-06-09

**Authors:** Yi Yang, Robert Sanford, Jun Yan, Gao Chen, Natalie L. Cápiro, Xiuying Li, Frank E. Löffler

**Affiliations:** a Key Laboratory of Pollution Ecology and Environmental Engineering, Institute of Applied Ecology, Chinese Academy of Sciences, Shenyang, Liaoning, China; b Department of Geology, University of Illinois at Urbana-Champaign, Urbana, Illinois, USA; c Center for Environmental Biotechnology, University of Tennessee, Knoxville, Tennessee, USA; d Department of Civil and Environmental Engineering, University of Tennessee, Knoxville, Tennessee, USA; e Department of Civil Engineering, Environmental Engineering Program, Auburn University, Auburn, Alabama, USA; f Department of Microbiology, University of Tennessee, Knoxville, Tennessee, USA; g Department of Biosystems Engineering and Soil Science, University of Tennessee, Knoxville, Tennessee, USA; h Biosciences Division, Oak Ridge National Laboratory, Oak Ridge, Tennessee, Tennessee, USA; i Joint Institute for Biological Sciences, Oak Ridge National Laboratory, Oak Ridge, Tennessee, USA; Lawrence Berkeley National Laboratory

**Keywords:** *Dehalococcoidia*, carbon cycling, hydrogen thresholds, organohalide respiration, syntrophy, thermodynamics

## Abstract

The class *Dehalococcoidia* within the *Chloroflexi* phylum comprises the obligate organohalide-respiring genera *Dehalococcoides*, *Dehalogenimonas*, and “*Candidatus* Dehalobium.” Knowledge of the unique ecophysiology and biochemistry of *Dehalococcoidia* has been largely derived from studies with enrichment cultures and isolates from sites impacted with chlorinated pollutants; however, culture-independent surveys found *Dehalococcoidia* sequences in marine, freshwater, and terrestrial biomes considered to be pristine (i.e., not impacted with organohalogens of anthropogenic origin). The broad environmental distribution of *Dehalococcoidia*, as well as other organohalide-respiring bacteria, supports the concept of active halogen cycling and the natural formation of organohalogens in various ecosystems. Dechlorination reduces recalcitrance and renders organics susceptible to metabolic oxidation by diverse microbial taxa. During reductive dechlorination, hydrogenotrophic organohalide-respiring bacteria, in particular *Dehalococcoidia*, can consume hydrogen to low consumption threshold concentrations (<0.3 nM) and enable syntrophic oxidation processes. These functional attributes and the broad distribution imply that *Dehalococcoidia* play relevant roles in carbon cycling in anoxic ecosystems.

## Abstract (precis)

The class *Dehalococcoidia* within the *Chloroflexi* phylum comprises the obligate organohalide-respiring genera *Dehalococcoides*, *Dehalogenimonas*, and “*Candidatus* Dehalobium.” Knowledge of the unique ecophysiology and biochemistry of *Dehalococcoidia* has been largely derived from studies with enrichment cultures and isolates from sites impacted with chlorinated pollutants; however, culture-independent surveys found *Dehalococcoidia* sequences in marine, freshwater, and terrestrial biomes considered to be pristine (i.

## INTRODUCTION

The phylum *Chloroflexi* is a deeply branching, highly heterogeneous bacterial lineage that currently comprises eight classes (*Dehalococcoidia*, *Anaerolineae*, *Caldilineae*, *Ktedonobacteria*, *Thermomicrobia*, *Ardenticatenia*, *Thermoflexia*, and *Chloroflexia*) with a diversity of phenotypes and lifestyles (1–3). This heterogeneity is also apparent in phylogenetic and comparative genome analyses, and the suggestion was made that the phylum *Chloroflexi sensu stricto* should only consist of the classes *Chloroflexia* and *Thermomicrobia*, while other members should be reclassified (4). In an effort to standardize bacterial taxonomy based on genome phylogeny, the reclassification of the *Chloroflexi* phylum (“*Chloroflexota*”) into eight classes (i.e., *Anaerolineae*, *Chloroflexia*, *Dehalococcoidia*, *Ktedonobacteria*, UBA2235, UBA4733, UBA5177, and UBA6077) was proposed (5).

**FIG 1:**
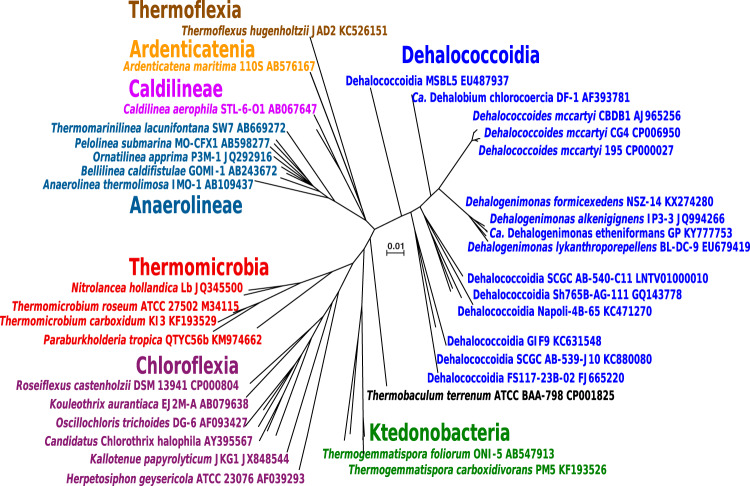
Phylogenetic tree of representative isolated and not-yet-cultured bacteria affiliated with the *Chloroflexi* phylum based on complete 16S rRNA gene sequences. Sequences were retrieved from the RDP database and imported into the Geneious software (Biomatters, Auckland, New Zealand) for alignment with MAFFT. The tree was constructed with Tree Builder using the default settings in Geneious. The scale bar indicates 0.01 nucleotide substitution per site.

Based on the SILVA database (release 138) (6) and the Ribosomal Database Project (RDP; release 11, update 5) (7), 8.5 and 3.1% of *Chloroflexi* 16S rRNA gene sequences, respectively, affiliate with the *Dehalococcoidia* class (Table 1). Currently, the *Dehalococcoidia* include two formally published genera, *Dehalococcoides* and *Dehalogenimonas*, and the candidate genus “*Dehalobium*,” all of which comprise obligate organohalide-respiring species (Fig. 1). All *Dehalococcoidia* in cultivation, including the *Dehalococcoides* and *Dehalogenimonas* isolates, can only derive energy from cleaving carbon-chlorine bonds (8, 9) although some may also use organobromine compounds as electron acceptors (10). While *Dehalococcoides* are strictly hydrogenotrophic (i.e., require hydrogen [H_2_] as an electron donor), at least some *Dehalogenimonas* species also utilize formate as an electron donor (9). Efforts in characterizing *Dehalococcoides* and *Dehalogenimonas* were largely driven by the interest in exploiting the dechlorination capabilities of these specialized bacteria for remediation of sites impacted with anthropogenic, chloroorganic pollutants such as chlorinated solvents (e.g., chlorinated ethenes). Examples of the utility of *Dehalococcoides* and *Dehalogenimonas* for environmental cleanup can be found in the recent comprehensive research update on organohalide-respiring bacteria and their applications (11).

**TABLE 1:** Numbers of 16S rRNA gene sequences representing *Chloroflexi*, including class *Dehalococcoidia* and unclassified *Chloroflexi*, and total bacteria in the Silva and RDP databases^*a*^

16S rRNA gene sequence database	No. (%) of *Chloroflexi* sequences			No. of total bacterial sequences
	*Dehalococcoidia*	Non-*Dehalococcoidia*	Unclassified	
RDP v11.5	1,411 (3.1)	30,328 (67.2)	13,419 (29.7)	3,196,041
RDP v11.5 isolates	48 (17.2)	213 (76.3)	18 (6.5)	515,987
RDP v11.5 uncultured	1,363 (3.0)	30,115 (67.1)	13,401 (29.9)	2,680,054
Silva SSU v138	16,297 (8.5)	176,547 (91.5)	30 (0.0)	8,394,436
Silva Ref SSU v138	2,654 (9.3)	25,837 (90.7)	5 (0.0)	1,916,743
Silva Ref NR SSU v138	1,601 (17.7)	7,444 (82.3)	4 (0.0)	381,662

a Included are sequences representing the existing taxonomy of the *Chloroflexi* phylum (i.e., *Dehalococcoidia* and non-*Dehalococcoidia Chloroflexi* sequences) and sequences that cannot be classified into the lower-rank taxonomy of the *Chloroflexi* phylum (i.e., unclassified *Chloroflexi*). Within the classified *Chloroflexi*, sequences belong to the *Dehalococcoidia* or any of the other seven non-*Dehalococcoidia* classes (i.e., *Anaerolineae*, *Caldilineae*, *Ktedonobacteria*, *Thermomicrobia*, *Ardenticatenia*, *Thermoflexia*, and *Chloroflexia*).

## DISTRIBUTION OF *DEHALOCOCCOIDIA* IN NATURE

Enrichment and isolation of *Dehalococcoidia* are challenging tasks. Information about the distribution of *Dehalococcoidia* in the environment relies on PCR-based surveys using specific primer sets and 16S rRNA gene amplicon, shotgun metagenome, and single-cell genome sequencing (see Table S1 in the supplemental material). An investigation of 116 uncontaminated terrestrial soil samples using a targeted PCR approach demonstrated that *Dehalococcoides*-like bacteria were present in the majority (>88%) of samples analyzed (12). A similar approach was used to study lake sediment samples from distinct locations (*n* = 20) across a geographic sulfur gradient, where 16S rRNA gene sequences of putative organohalide-respiring *Dehalococcoidia* were found in 67 out of 68 samples examined (13). Shotgun metagenome and 16S rRNA gene amplicon sequencing identified a diversity of *Dehalococcoidia* in samples collected from various environments, including marine subsurface sediments (14–21). For example, bacterial 16S rRNA gene sequences affiliated with the *Chloroflexi* phylum dominated in shallow sediment 9.5 to 364 meters below the seafloor (mbsf) and became rare in deeper sediment (1,372 mbsf) (17). Closer scrutiny of the shallow marine sediment sequences retrieved from the DNA Data Bank of Japan (DDBJ accession number DRA001030) using the SILVAngs pipeline (6) revealed that more than 95% of all *Chloroflexi* sequences classify within the *Dehalococcoidia* class. Members of the *Dehalococcoidia* formed a dominant group in sediment samples from the eastern Juan de Fuca Ridge flank, a well-studied hydrothermal environment (22). Another metagenomic study reported abundant *Dehalococcoidia* sequences (up to 14.2% of all reads) in a methane (CH_4_) hydrate-bearing sediment sample from the Ulleung Basin in the East Sea of Korea (23). *Dehalogenimonas* sequences were present in 60 m depth in a perennially stratified Arctic lake with saline (2.9 to 3.2%), anoxic bottom waters (24). Phylogenetically diverse *Dehalococcoidia* sequences were found at different depths in anoxic waters of the remote meromictic Lake Pavin, with 11 out of 65 nearly complete 16S rRNA gene sequences showing 80 to 98% nucleotide identity with *Dehalococcoides* or *Dehalogenimonas* (25). *Dehalogenimonas* 16S rRNA gene sequences were also detected in the photic layer (0 to 25 m) and the near-bottom zone (1,465 to 1,515 m) of Lake Baikal, the world’s deepest and most voluminous lake (20). No deep-sea sediment *Dehalococcoidia* have yet been cultured and isolated; however, single cell sequencing generated several incomplete *Dehalococcoidia* genomes (26–30). Reductive dehalogenase (RDase) genes, which encode the key enzymes for organohalide respiration, have been detected in various marine subsurface sediments (e.g., Juan de Fuca Ridge Flank, Nankai Trough Forearc Basin) (31, 32) but have not (yet) been found on published single-cell *Dehalococcoidia* genomes, suggesting that deep-sea sediment *Dehalococcoidia* may have the potential to utilize energy metabolisms other than organohalide respiration (26–30).

10.1128/mSystems.00757-19.1 TABLE S1 List of *Dehalococcoidia* identified in various contaminated and pristine ecosystems. Included in the table are isolates and uncultured *Dehalococcoidia* identified in mixed cultures and environmental samples. The light-blue background indicates *Dehalococcoidia* isolates analyzed by whole-genome sequencing. Single-cell genome sequencing (light-gray background), 16S rRNA gene amplicon sequencing (light-yellow background), and metagenome sequencing (light-green background) were applied to retrieve *Dehalococcoidia* sequence information from mixed cultures and environmental samples from various geographic locations. Download Table S1, XLSX file, 0.03 MB. Copyright © 2020 Yang et al. 2020 Yang et al. This content is distributed under the terms of the Creative Commons Attribution 4.0 International license.

## ROLES OF *DEHALOCOCCOIDIA* IN CONTAMINATED AND PRISTINE ENVIRONMENTS

The need for cost-effective remediation of sites impacted with chlorinated contaminants was a driver leading to the discovery of *Dehalococcoidia* capable of utilizing chlorinated organic compounds as respiratory electron acceptors (33, 34). The organohalide respiration process revolutionized bioremediation at sites impacted with chlorinated pollutants (35, 36), and detailed characterization work transformed our understanding of the physiology, biochemistry, and evolution of organohalide-respiring *Dehalococcoidia* (11). What is generally not acknowledged is that most organochlorines are not only xenobiotics (i.e., anthropogenic chemicals) but also have natural origins. Natural organohalogens commonly occur in many undisturbed (i.e., pristine) environments. More than 5,000 naturally occurring organohalogens have been reported to date, the majority of which are organochlorines (11, 37–39) that *Dehalococcoidia* potentially can utilize as electron acceptors (40). Chlorinated organics are produced in biotic and abiotic processes in various aquatic and terrestrial environments. The diversity of naturally occurring organochlorines may explain the astounding diversity of genes with predicted RDase function in *Dehalococcoidia* genomes. As of March 2020, the NCBI Identical Protein Groups database lists 842 distinct *Dehalococcoides* and 219 distinct *Dehalogenimonas* putative RDase proteins, but the substrate specificity (i.e., the range of chlorinated electron acceptors used) has only been assigned to a small subset. Of note, *Dehalococcoidia* possess RDase genes and also dehalogenate truly anthropogenic chemicals with no evidence of natural formation, such as polychlorinated biphenyls (PCBs) (41). A possible explanation is that RDases with broad substrate range dechlorinate anthropogenic PCBs. Intriguing is the possibility of evolutionary processes that allow RDase genes of *Dehalococcoidia* to adapt to chlorinated xenobiotics in fairly short time periods (i.e., the production of PCBs commenced in the 1930s). In support of this hypothesis, the dynamic rearrangement and mobilization of RDase genes in *Dehalococcoides* and *Dehalogenimonas* genomes have been observed (42–45).

In pristine environments, *Dehalococcoidia* may perform reductive dehalogenation by utilizing natural organochlorines as electron acceptors. The removal of chlorine atoms transforms recalcitrant organochlorines into compounds that can then be metabolized and mineralized by other microorganisms. Although nature produces a plethora of organochlorines, these compounds do not accumulate and evade detection, presumably because microorganisms, such as *Dehalococcoidia*, reduce recalcitrance by breaking carbon–chlorine bonds. The absence of widespread elevated concentrations of natural organochlorines supports the notion that nature evolved a balanced system of organochlorine production and consumption. Because the mass of organochlorines in pristine environments is generally low, the natural formation of organochlorines often goes unnoticed; however, the flux of carbon through halogenation-dehalogenation cycles can be substantial. Extensive carbon turnover through halogenation-dehalogenation cycles has been demonstrated in a few natural (46–48) and engineered ecosystems (49), but accurate and comprehensive flux measurements that could help estimate the total amounts of chlorinated electron acceptors turned over in various environmental systems are largely lacking. Of note, the cultivated *Dehalococcoidia* can only grow via organohalide respiration without any other demonstrated growth-supporting energy metabolism. Therefore, the distribution of *Dehalococcoidia* in various environmental systems supports the hypothesis that natural halogen cycling (i.e., the natural formation and consumption of halogenated electron acceptors) occurs broadly. The following examples illustrate relevant ecological roles organohalide-respiring *Dehalococcoidia* can fulfill in pristine environments.

## *DEHALOCOCCOIDIA* ENABLE SYNTROPHIC PROCESSES INCLUDING THE ANAEROBIC OXIDATION OF METHANE

Reductive dechlorination is an electron-consuming process, and H_2_ fulfills the electron donor demand of many organohalide-respiring bacteria, including the *Dehalococcoidia*. Under standard conditions (i.e., solutes at 1 M concentration and gases at 1 atmosphere partial pressure) at pH 7, reductive dechlorination is associated with Gibb’s free energy changes (Δ*G*°′) in the range of −127 to −193 kJ/mol of H_2_ (50, 51). These Δ*G*°′ values indicate more energy is gained in the organohalide respiration process than in sulfate reduction (−38 kJ/mol of H_2_), ferric iron reduction (−44.6 kJ/mol of H_2_ with amorphous hydrous ferric oxide), CO_2_ reduction to CH_4_ (i.e., methanogenesis, −33.9 kJ/mol of H_2_), or CO_2_ reduction to acetate (i.e., reductive acetogenesis, −26.1 kJ/mol of H_2_) and is in the range of nitrate reduction to nitrite (−158.1 kJ/mol of H_2_) (Table 2).

**TABLE 2:** Gibbs free energy changes under standard conditions (i.e., 298.15 K [25°C], pH 7.0), concentrations of solutes at 1 M and partial pressures of gases at 1 atmosphere) for AOM and different H_2_-consuming redox reactions^*a*^

No.	Redox reaction	Gibbs free energy change (Δ*G*°′) in kJ/mol of:			Redox potential E°′ (V)^*b*^	Theoretical H_2_ consumption threshold concn (nM)^*c*^	Experimentally determined H_2_ threshold concn (nM)^*d*^
		H_2_	Electron acceptor	Electrons			
1	CH_4_(g) + 3H_2_O → HCO_3_^–^ + 4H_2_(g) + H^+^	33.9	135.4	16.9	–0.18	NA	NA
2	2HCO_3_^–^ + 4H_2_(g) + H^+^→ acetate^–^ + 4H_2_O	–26.1	–52.1	−13.0	0.13	863.0	>100
3	HCO_3_^–^ + 4H_2_(g) + H^+^ → CH_4_(g) + 3H_2_O	–33.9	−135.4	–16.9	0.18	162.3	12.4–13.6
4	SO_4_^2–^ + 4H_2_(g) + 2H^+^ → H_2_S(aq) + 4H_2_O	–38.0	–151.9	–19.0	0.20	15.4	1–15
5	2Fe(OH)_3_(s) + 4H^+^ + H_2_(g) → 2Fe^2+^(aq) + 6H_2_O	–44.6	–22.3	–22.3	0.23	6.06 × 10^–10^	0.1–0.8
6	NO_3_^–^ + H_2_(g) → NO_2_^–^ + H_2_O	–158.1	–158.1	–79.0	0.82	5.39 × 10^–22^	0.026–0.036
7	PCE(aq) + H_2_(g)→ TCE(aq) + H^+^ + Cl^–^	–173.3	–173.3	–86.7	0.90	7.19 × 10^–25^	<0.3

a Also indicated are H_2_ consumption threshold concentrations associated with H_2_-consuming redox reactions. The Δ*G*°′ values for all reactions except reductive dechlorination of tetrachloroethene (PCE) to trichloroethene (TCE) were calculated using Geochemists WorkBench (GWB14). The Δ*G*°′ value for reductive dechlorination was calculated according to values obtained from Dolfing and Janssen (51). NA, not applicable; (g), gas phase; (aq), aqueous phase; (s), solid.

b Redox potentials and Gibbs free energy changes were calculated according to Δ*G*°′ = –*n*FΔE°′ is the number of moles of electrons transferred in the reaction, and F is the Faraday’s constant (96.5 kJ/V). Redox reactions are numbered in the first column of the table as follows: 1, AOM; 2, H_2_/CO_2_ reductive acetogenesis; 3, methanogenesis; 4, sulfate reduction; 5, ferric iron reduction; 6, nitrate reduction; and 7, organohalide respiration.

c The theoretical H_2_ consumption threshold concentrations were calculated based on equation 6 using a Δ*G′* = −10 kJ/mol H_2_ and assumed standard conditions for all other reactants (1 M for solutes and 1 atm for gases).

d Shown are experimentally determined H_2_ threshold concentrations reported in the literature (51, 52, 55, 58, 59, 66, 81, 82). The measured data are substantially higher than the theoretical values because the available analytical instrumentation cannot measure H_2_ reliably below 50 ppb (∼0.04 nM).

In contrast to these electron-accepting, formally H_2_-consuming processes, the anaerobic oxidation of methane (AOM) is an electron-donating, formally H_2_-forming process with unfavorable energetics under standard conditions (equation 1).

(1) $${\text{CH}}_{\text{4}}+{\text{ 3H}}_{\text{2}}\text{O }\to {\text{ HCO}}_{\text{3}}{}^{-}+{\text{ 4H}}_{\text{2}}+{\text{ H}}^{+}\;\Delta G^{\text{o}}\prime =+\text{135.4 kJ}/{\text{mol CH}}_{\text{4}}$$

The energetics of AOM become favorable (i.e., negative Δ*G* values) when the H_2_ (i.e., reducing equivalents) generated during CH_4_ oxidation is consumed in an associated electron-accepting, H_2_-consuming process.

To calculate H_2_ consumption threshold concentrations and Gibbs free energy changes associated with different redox reactions, equations 2 to 6 were used, where R is the ideal gas constant (8.314 J × K^−1^ × mol^−1^), T is the temperature (298 K), Δ*G*°′ is the free-energy change at pH 7 under standard conditions, Δ*G′* is the free-energy change at pH 7 under nonstandard concentration conditions, “*a”* refers to the activity (i.e., the effective concentration) of the different chemicals in the reaction, and EA refers to the electron acceptor. The letters a, b, c and d are stoichiometric coefficients for reactants EA, H_2_, C and D, respectively.

(2) $$\Delta G\prime =\Delta G^{\text{o}}\prime +\mathrm{RTln}(\frac{(a_{{\mathrm{HCO}}_{3}^{-}}){\left(a_{H_{2\;}}\right)}^{4}}{a_{{\mathrm{CH}}_{4\;}}})$$

(3) $$a_{{\text{H}}_{2}}={\left(\frac{\left(a_{{\text{CH}}_{4}}\right)}{\left(a_{{\text{HCO}}_{3}^{-}}\right)}\text{exp}\left(\frac{\Delta G\prime -\Delta G^{\text{o}}\prime }{\text{RT}}\right)\right)}^{1/4}$$

(4) $$\text{aEA}+{\text{bH}}_{2}\to \text{cC}+\text{dD}$$

(5) $$\Delta G\prime =\Delta G^{\text{o}}\prime +\text{RTIn}\left(\frac{{\left(a_{C}\right)}^{c}{\left(a_{D}\right)}^{d}}{{\left(a_{H_{2}}\right)}^{b}{\left(a_{EA}\right)}^{a}}\right)$$

(6) $$a_{{\text{H}}_{2}}={\left(\frac{{\left(a_{\text{C}}\right)}^{c}{\left(a_{\text{D}}\right)}^{d}}{{\left(a_{\text{EA}}\right)}^{a}}*\frac{1}{\text{exp}\left(\frac{\Delta G\prime -\Delta G^{\text{o}}\prime }{\text{RT}}\right)}\right)}^{1/b}$$

To allow energy conservation in anaerobic microorganisms, it has been argued that the Gibbs free energy change (i.e., Δ*G*) of the reaction must be more negative than −10 ± 1 kJ/mol (53, 54). Based on this assumption, any H_2_ concentration exceeding 29.7 μM (3,888 Pa) would render AOM unfavorable (i.e., Δ*G′* > −10 ± 1 kJ/mol) and would not allow microorganisms capable of AOM to harvest energy from the process. The breaking point H_2_ concentration of 29.7 μM is based on presumed environmentally relevant concentrations of reactants and products (i.e., [CH_4_] = 2.14 atm, [HCO_3_^–^] = 20.2 mM) and a Δ*G*′ of −10 kJ/mol, and was calculated according to equations 2 and 3 (52). These calculations assumed that *a* values approximate the concentration of HCO_3_^–^ and partial pressure of CH_4_. Partial pressures were entered directly into the calculations as indicated in Table 2, and the equilibrium aqueous phase concentrations of CH_4_ and H_2_ were calculated using the respective Henry’s constant.

**FIG 2:**
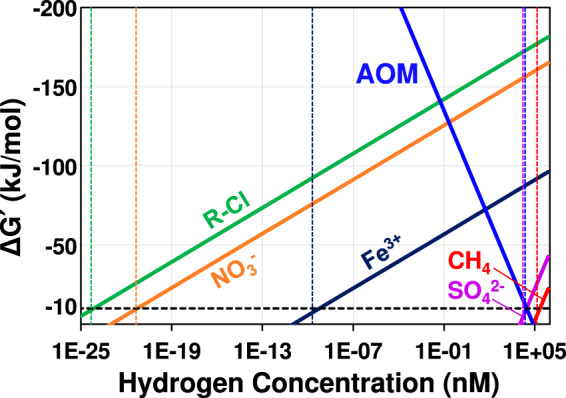
Visualization of Δ*G′* values calculated per mol of electron acceptor consumed for different redox reactions versus the H_2_ concentration. Depicted are the redox reactions 3 to 7 listed in Table 2. The Δ*G′* values were calculated according to equation 6 except for AOM, which was calculated according to equation 3 (see the text). The minimum Gibbs free energy yield allowing an anaerobic microbe to conserve energy falls in the range of −10 ± 1 kJ/mol for each reaction (horizontal, dashed black line). At Gibbs free energy changes greater (i.e., more positive) than −10 ± 1 kJ/mol, microorganisms cannot conserve energy and cease metabolic activity (53). The concentrations of chemical species representative of environments favoring sulfate reduction, ferric iron reduction, nitrate reduction, and organohalide respiration were retrieved from published data (53, 79) and the Substance Priority List database (www.atsdr.cdc.gov/spl/resources/index.html). The following concentrations were used for calculating Δ*G′* at different H_2_ concentrations: HCO_3_^–^, 2.020 × 10^−2^ M; CH_4_, 3.000 × 10^−5^ M; SO_4_^2–^, 5.200 × 10^−3^ M; H_2_S, 8.000 × 10^−4^ M; Fe^2+^, 3.000 × 10^−5^ M; NO_3_^–^, 1.850 × 10^−4^ M; NO_2_^–^, 1.160 × 10^−5^ M; PCE, 3.190 × 10^−7^ M; TCE, 7.290 × 10^−7^ M; Cl^–^, 1.690 × 10^−2^ M; acetate, 1.000 × 10^−4^ M; cDCE, 4.570 × 10^−7^ M. The headspace partial pressures of CH_4_ and H_2_ were converted to aqueous concentration units (M) using Henry’s law constants of 1.4 × 10^−5^ for CH_4_ and of 7.8 × 10^−6^ for H_2_ (80, 82). The dashed vertical lines indicate the theoretical H_2_ consumption threshold concentrations for each reaction. The abbreviations CH_4_, SO_4_^2–^, Fe^3+^, NO_3_^–^, and R-Cl indicate different redox processes utilizing H_2_ as the electron donor, including methanogenesis (reaction 3 in Table 2), sulfate reduction (reaction 4), iron reduction (reaction 5), nitrate reduction to nitrite (reaction 6), and reductive dechlorination (reaction 7), respectively.

In anoxic environments, the H_2_ partial pressure is the primary thermodynamic control determining the extents and rates of syntrophic oxidation processes and carbon turnover (52). AOM has been observed in anoxic marine sediments and is mediated by a syntrophic partnership between anaerobic methanotrophic archaea (ANME) and sulfate-reducing bacteria (55, 56). The candidates for syntrophic partners of ANME have been extended to ferric iron-reducing microorganisms (57, 58), and various mechanisms, including interspecies H_2_ transfer and direct interspecies electron transfer, may enable these syntrophic partnerships (59–61). An interesting observation was the partner population-independent AOM in nitrate-reducing cultures comprising “*Candidatus* Methanoperedens nitroreducens” and in nitrite-reducing cultures comprising the NC10 bacterium “*Candidatus* Methylomirabilis oxyfera” (62). Since both organisms are not available in pure culture, possible syntrophic partnerships with nitrate/nitrite-reducing populations cannot be ruled out, and it remains to be seen if single organisms can carry out AOM (63, 64). In interspecies H_2_ transfer, hydrogenotrophic partner populations (e.g., sulfate-, ferric iron-, and nitrate-reducing microorganisms) consume the H_2_ produced from the oxidation of CH_4_ to low H_2_ partial pressures and create conditions with favorable thermodynamics for AOM to proceed (59, 65) (Table 3). On grounds of thermodynamics, reductive dehalogenation in partnership with AOM yields greater free energy changes compared to sulfate reduction (Table 3) (51, 55, 66). The theoretical H_2_ consumption threshold concentration for sulfate reduction under relevant *in situ* concentrations of reactants and products (i.e., Δ*G′* values) is about 15.4 nM (∼2 Pa) (see Fig. 2 for details). By comparison, reductive dechlorination, nitrate reduction, and ferric iron reduction under environmentally relevant concentrations can theoretically maintain much lower H_2_ consumption threshold concentrations of 7.19 × 10^−25^, 5.39 × 10^−22^, and 6.06 × 10^−10 ^nM, respectively, indicating these processes would be far better partners for enabling syntrophic processes (Table 2). Organohalide-respiring *Dehalococcoidia* could serve as a relevant H_2_ sink and maintain H_2_ concentrations far below the 29.7 μM value needed to enable AOM (67) (Fig. 2). The syntrophic partnership between organohalide-respiring *Dehalococcoidia* and ANME could be a relevant function of *Dehalococcoidia* in various anoxic environmental systems with CH_4_ and a sustained formation of organohalogens. In support of this hypothesis, a recent study demonstrated the coexistence of *Dehalococcoides* and ANME in a permanently ice-covered Antarctic lake (21), suggesting a possible linkage between organohalide respiration and AOM *in situ*. To summarize, organohalide-respiring *Dehalococcoidia* would be ideal syntrophic partners for energetically challenging, syntrophic processes, such as AOM (Table 3), and are likely relevant drivers of carbon turnover and nutrient cycling in environments where other electron acceptors are absent and active production of organochlorines occurs.

**TABLE 3:** Gibbs free energy changes (Δ*G*) associated with anaerobic CH_4_ oxidation coupled to the reduction of different electron acceptors under standard conditions (Δ*G*°′ values) and environmentally relevant concentrations of reactants and products (Δ*G′* values)^*a*^

Redox reaction	Δ*G*°′, kJ/mol of:			Δ*G′*, kJ/mol of:		
	CH_4_	EA	Electrons	CH_4_	EA	Electrons
CH_4_(g) + SO_4_^2–^ + H^+^ → HCO_3_^–^ + H_2_S(aq) + H_2_O	–16.5	–16.5	–2.06	–5.0	–5.0	−0.63
CH_4_(g) + 8Fe(OH)_3_(s) + 15H^+^ → HCO_3_^–^ + 8Fe^2+^ + 21H_2_O	46.2	5.78	5.78	–144.1	–18.0	–18.0
CH_4_(g) + 4 2-CP(aq) + 3H_2_O → HCO_3_^–^ + 4 phenol(aq) + 5H^+^ + 4Cl^–^	–476.3	–119.1	−59.5	–486.2	–121.6	–60.8
CH_4_(g) + 4 MCB(aq) + 3H_2_O → HCO_3_^–^ + 4 benzene(aq) + 5H^+^ + 4Cl^–^	–477.9	–119.5	–59.7	–499.3	–124.8	–62.4
CH_4_(g) + 4NO_3_^–^ → HCO_3_^–^ + 4NO_2_^–^ + H_2_O + H^+^	–510.7	–127.7	–63.8	–501.4	–125.4	–62.7
CH_4_(g) + 4 PCE(aq) + 3H_2_O → HCO_3_^–^ + 4TCE(aq) + 5H^+^ + 4Cl^–^	–557.9	–139.5	–69.7	–574.0	–143.5	–71.8
CH_4_(g) + 4 TCE(aq)+ 3H_2_O → HCO_3_^–^ + 4 cDCE(aq) + 5H^+^ + 4Cl^–^	–539.9	–135.0	–67.5	–568.8	–142.2	–71.1
CH_4_(g) + 4 1,2-DCA(aq) + 2H_2_O → HCO_3_^–^ + 4 ethene(aq) + 9H^+^ + 8Cl^–^	−853.5	–213.4	−106.7	–839.5	−209.9	−104.9

*^a^*The Δ*G*°′ values for all reactions except reductive dechlorination of tetrachloroethene (PCE) to trichloroethene (TCE) were calculated using Geochemists WorkBench (GWB14). The Δ*G*°′ for reductive dechlorination was calculated according to Dolfing and Janssen (51). The Δ*G′* values were calculated according to equation 5 (see the text) with CH_4_ substituting for H_2_ and using the concentration values listed below. Abbreviations: PCE, tetrachloroethene; TCE, trichloroethene; cDCE, *cis*-1,2-dichloroethene; 1,2-DCA, 1,2-dichloroethane; MCB, monochlorobenzene; 2-CP, 2-chlorophenol; EA, electron acceptor; (g), gas phase; (aq), aqueous phase; (s), solid. The following concentrations were used for calculating Δ*G′* values: HCO_3_^–^, 2.020 × 10^−2^ M; CH_4_, 3.000 × 10^−5^ M; SO_4_^2–^, 5.200 × 10^−3^ M; H_2_S, 8.000 × 10^−4^ M; Fe^2+^, 3.000 × 10^−5^ M; NO_3_^–^, 1.850 × 10^−4^ M; NO_2_^–^, 1.160 × 10^−5^ M; PCE, 3.190 × 10^−7^ M; TCE, 7.290 × 10^−7^ M; Cl^–^, 1.690 × 10^−2^ M; acetate, 1.000 × 10^−4^ M; cDCE, 4.570 × 10^−7^ M; DCA, 4.730 × 10^−7^ M; ethene, 1.340 × 10^−3^ M; 2-CP, 5.072 × 10^−7^ M; phenol, 2.178 × 10^−6^ M; MCB, 5.313 × 10^−7^ M; benzene, 7.105 × 10^−7^ M.

## *DEHALOCOCCOIDIA* BIOMASS FUELS MICROBIOMES IN HIGHLY OLIGOTROPHIC ENVIRONMENTS

Organohalide-respiring *Dehalococcoidia* use H_2_ at very low concentrations and are active in anoxic environments where organochlorines form naturally. *Dehalococcoidia* generate approximately four times more biomass per mole of H_2_ oxidized than methanogens (50). When hydrolysis and fermentation reactions sustain elevated H_2_ concentrations, hydrogenotrophic methanogenesis and organohalide respiration can cooccur, as long as chlorinated electron acceptors are available. In highly oligotrophic environments (e.g., the energy-deprived deep subsurface) the formation of H_2_ is thermodynamically controlled, and hydrogenotrophic organohalide-respiring *Dehalococcoidia* can outcompete other H_2_-consuming microorganisms (e.g., methanogens and sulfate reducers) for electron donor. In environments where energy sources limit microbial metabolism and growth (e.g., the deep subsurface), *Dehalococcoidia* necromass could be a relevant source of nutrients and energy, suggesting that *Dehalococcoidia* play relevant functions in carbon and energy cycling and support microbiomes in energy-depleted, oligotrophic ecosystems. This process likely extends to environments without measurable pools of chlorinated organics because balanced chlorination and dechlorination reactions maintain low steady-state organochlorine concentrations but provide sufficient flux to sustain *Dehalococcoidia* populations.

## *DEHALOCOCCOIDIA* MEDIATE RECALCITRANCE OF ORGANIC COMPOUNDS AND AN ENVIRONMENTAL SYSTEM’S CHLORINE STORAGE CAPACITY

Natural chlorination is a widespread phenomenon in many environmental systems (40) and enhances the recalcitrance of organic compounds. Chlorination also influences the environmental phase partitioning and transport of organic compounds and increases the chlorine retention time, which impacts ecosystem processes, as documented in forest soils and marine sediments (46, 47, 68). The cleavage of carbon-chlorine bonds by abiotic and biotic processes releases inorganic chloride and transforms recalcitrant carbon into bioavailable and biodegradable organic carbon that various microbial taxa can metabolize (47). Thus, organohalide-respiring *Dehalococcoidia* play key roles by removing recalcitrance and rendering the remaining nonhalogenated organics (or with a lower degree of chlorination) more susceptible to microbial metabolism. Inorganic chloride is water-soluble and prone to leaching, especially in well-drained soils experiencing elevated rainfall. Chloride is an essential plant micronutrient and extensive chloride loss can affect plant productivity. Thus, the fine-tuned interplay between chlorination and dechlorination reactions impacts soil microbiomes, as well as macroflora, with potentially far-reaching consequences for plant productivity and carbon and nutrient cycling. Such cross-kingdom interactions involving halogen cycling are poorly understood and organohalide-respiring bacteria may play a crucial role in balancing this system.

## *DEHALOCOCCOIDIA* ATTENUATE SIGNALING MOLECULES AND CHEMICAL DETERRENTS

Antibiotics selectively kill or inhibit competing (micro)organisms or have signaling function, and this weapon versus signal debate has received considerable attention (69–73). Antibiotics at low, subinhibitory concentrations may serve signaling purposes and can impact bacterial virulence, biofilm formation, quorum sensing, gene expression, and gene transfer (73). Many natural (e.g., vancomycin) and anthropogenic (e.g., triclosan) antibiotics are chlorinated organic compounds (74). An increasing number of halogenated natural products have been identified and characterized as chemical repellents, feeding deterrents and signaling molecules, including chlorinated steroid glycosides from the German cockroach (*Blattella germanica*), brominated organic compounds (e.g., bromoindoles, bromophenols, and bromopyrroles) from various marine sponges (e.g., *Aplysina aerophoba*, *Agelas wiedenmayeri*, and *Agelas conifera*), and brominated diterpenes from the marine red alga *Laurencia saitoi* (37, 39, 75–77). *Dehalococcoidia* may interact with organohalogen-producing organisms and play a role in recycling halogenated antibiotics and signaling molecules. For instance, the bacterial community associated with *Aplysina aerophoba*, a marine sponge that produces organobromine compounds, has been demonstrated to dehalogenate halogenated phenolic compounds, and a 16S rRNA gene sequence closely related to *Dehalococcoidia* was recovered from the sponge-associated microbiota (76, 77). Thus, *Dehalococcoidia* may play crucial ecological roles in disarming chemical defense systems and have functions as signal-attenuating agents by dehalogenating halogenated signaling molecules and antibiotics.

## KNOWLEDGE GAPS

### Innovative strategies for cultivating not-yet-cultured *Dehalococcoidia*.

Diverse uncultured *Dehalococcoidia* have been identified in various sequencing studies (i.e., shotgun metagenomic sequencing, amplicon sequencing, single-cell sequencing) (see Table S1 in the supplemental material). Single-cell sequencing has expanded the *Dehalococcoidia* pangenome, but the approach is plagued by cell selection biases and incomplete genome information (28). Cultivation from single cells has not been successful, preventing detailed physiological and biochemical studies of *Dehalococcoidia* captured by single-cell sorting approaches (26, 27, 78). The hope is that the increasing information obtained from metagenome and single-cell genome sequencing will guide innovative cultivation and enrichment strategies to obtain cultures for detailed physiological and biochemical studies.

### Quantifying the roles of *Dehalococcoidia* in syntrophy and carbon cycling.

Hydrogenotrophic organohalide-respiring *Dehalococcoidia* consume H_2_ to low consumption threshold concentrations and are ideal partners in syntrophic processes, such as AOM, that are thermodynamically controlled by the prevailing H_2_ partial pressure. The concentrations (i.e., pools) of organochlorines in pristine environments are typically low; however, very little is known about the *in situ* rates of halogenation and dehalogenation reactions. Thus, the fluxes between nonhalogenated and halogenated pools of organic material may be large and the impact of hydrogenotrophic organohalide-respiring bacteria for carbon cycling may be far greater than is currently acknowledged. Therefore, concerted efforts are needed to measure fluxes of organohalogens in pristine environments, so that the role of organohalide-respiring bacteria for syntrophic processes can be more clearly understood and delineated. To advance the understanding of the dynamics of *Dehalococcoidia* populations in different microbiomes, time-resolved absolute quantitative information should be obtained. Should further research corroborate that *Dehalococcoidia* are crucial partner populations for syntrophic processes such as AOM, or key specialist degraders of halogenated signaling molecules, the resilience of *Dehalococcoidia* to changing environmental conditions (e.g., pH, warming temperatures, decreased or increased soil moisture content, and land-use change) must be explored, so that ecosystem-level responses can be predicted.

### Biotechnological applications.

If the anticipated progress in cultivation can be realized and a broader suite of cultures and isolates from diverse environments becomes available, the roles of *Dehalococcoidia* in carbon and nutrient cycling in natural, pristine and organohalogen degradation in contaminated environments can be elucidated. Such efforts will likely lead to the discovery of dechlorinators with novel RDase genes, which can advance monitoring regimes and treatment of sites impacted with anthropogenic chlorinated contaminants. Organohalide-respiring *Dehalococcoidia* harbor a sizeable genetic diversity of RDase genes, but comparably few substrates have been identified, suggesting that *Dehalococcoidia* can utilize a much broader range of organohalogens as electron acceptors than is currently appreciated. Largely lacking is kinetic information about RDases (i.e., the affinity to chlorinated electron acceptors [*K_m_* values] and the turnover rates), which would be necessary to gauge the contributions of *Dehalococcoidia* for transforming halogenated micropollutants (e.g., pesticides, pharmaceuticals like chloroquine and hydroxychloroquine). Bioaugmentation with *Dehalococcoides*-containing consortia has promoted *in situ* contaminant removal at a large number of sites (11); however, bioremediation is still an empirical practice and comprehensive knowledge of the diversity and ecophysiology of organohalide-respiring *Dehalococcoidia* can advance the approach to a science with predictable outcomes.
